# Genomic Characterization and Putative Recombination Signals of Ostrich-Associated Parvovirus in South China

**DOI:** 10.3390/microorganisms14102168

**Published:** 2026-09-25

**Authors:** Yangyan Yin, Yongcui Feng, Leping Wang, Changting Li, Chunping Tan, Xian Li, Zihao Wang, Binhua Wen, Yanwen Zhang, Hao Peng

**Affiliations:** 1 Guangxi Key Laboratory of Animal Breeding, Disease Control and Prevention, College of Animal Science and Technology, Guangxi University, Nanning 530004, China; 15177906141@163.com; 2 Guangxi Key Laboratory of Veterinary Biotechnology, Guangxi Veterinary Research Institute, Nanning 530001, China; kkdfyc@163.com (Y.F.);; 3 College of Food and Quality Engineering, Nanning University, Nanning 530299, China; 4 College of Animal Science and Technology, Guangxi Agricultural Engineering Vocational Technological College, Nanning 530028, China; 5 College of Animal Science and Technology, Guangxi Vocational University of Agriculture, Nanning 530007, China

**Keywords:** cross-species transmission, ostrich, paralysis, parvovirus, recombination analysis

## Abstract

Since 2020, a disease characterized by paralysis and diarrhea has affected ostriches in southern China, resulting in significant economic losses for ostrich farms. Initial analyses of clinical samples from affected birds indicated the presence of a parvovirus as a potential causative agent. This study aimed to identify and characterize the involved pathogenic parvovirus, analyze its genetic features, determine its phylogenetic relationship with known parvovirus strains, and investigate its role in the observed clinical symptoms. We collected 15 clinical samples ((including sciatic nerve, spleen, kidney, and other visceral organs) from 15 affected ostriches and performed gross pathological and histopathological examinations. Whole-genome sequencing of the detected ostrich parvovirus (OsPV) was conducted, followed by phylogenetic, genetic distance, and recombination analyses. Ostrich parvovirus (OsPV) was detected in the collected clinical samples, and viral genomic DNA was identified across multiple tissues, indicating broad tissue distribution. Phylogenetic analysis revealed that OsPV forms a unique branch within the goose parvovirus (GPV) group and is closely related to Chinese GPV strains, particularly the DY16 strain. Notably, the identified virus possessed two 14 bp deletions in its inverted terminal repeats. Furthermore, the virus (designated GX-2020) was identified as carrying recombination signals derived from the SYG61v and B strains of GPV. Recombination among classical GPV strains likely facilitated the emergence of OsPV and may point to possible cross-species transmission or host-range expansion of GPV. This study offers new insights into the evolution, molecular biology, and host adaptation of parvoviruses in China.

## 1. Introduction

The Parvoviridae family consists of small, non-enveloped, single-stranded DNA viruses divided into two subfamilies: Parvovirinae (infecting vertebrates) and Densovirinae (infecting invertebrates) [1,2]. These viruses exhibit a broad host range, with ancestors originating tens to hundreds of millions of years ago and subsequently spreading widely across both vertebrates and invertebrates [3,4]. Recent studies have demonstrated that parvoviruses are widely circulating among various bird species globally, significantly expanding their known diversity and host range. This spread has negatively impacted the poultry industry, causing substantial economic losses [5,6]. Another aspect that has drawn attention to avian parvoviruses is their propensity for recombination among strains, a process that significantly enhances their evolutionary potential [7]. Furthermore, the increasing availability of parvovirus sequences in public databases has uncovered numerous previously unknown virus–host relationships [8]. For instance, studies has identified a novel duck parvovirus (NDPV), recognized as a new variant of goose parvovirus (GPV) that induces short beak and dwarfism syndrome (SBDS) in Cherry Valley ducks and mule ducks with high morbidity and low mortality in mainland China [9,10,11]. NGPV infection has also been reported in Egypt, Poland, Vietnam and other countries [12,13,14]. Meanwhile, research have demonstrated that novel recombinant Muscovy duck parvoviruses (rMDPV), generated by recombination between Muscovy duck parvovirus (MDPV) and goose parvovirus (GPV) which both belong to the Dependoparvovirus genus within the Parvovirinae subfamily, are prevalent in waterfowl in China and are associated with high morbidity and mortality [15,16,17,18].

The ostrich is the largest living bird and belongs to the ratite family of birds, and ostrich farming is an economic activity worldwide [19]. To date, avian parvovirus research has predominantly focused on species such as geese, Muscovy ducks, chickens, and turkeys, with limited studies on ostriches [20,21,22]. In China, ostrich parvovirus infection was first reported in 2020 following outbreaks of a disease characterized by paralysis in ostriches aged 1 to 4 months in Beijing, Hebei, Shanxi, and Yunnan provinces, with morbidity rates ranging from 30% to 80%. A novel ostrich parvovirus (OsPV) was detected in these affected chicks, and subsequent whole-genome sequencing and phylogenetic analysis confirmed its molecular evolutionary relationship with GPV and MDPV [23].

In this study, we report the identification of a paralytic disease in ostriches with a morbidity rate exceeding 80% in southern China. Using clinical samples from affected and deceased ostriches, we systematically analyzed the genetic characteristics and evolutionary patterns of ostrich parvovirus (OsPV). This research focuses on investigating the molecular features of OsPV to provide theoretical evidence for the cross-species transmission and genetic recombination of goose parvovirus.

## 2. Materials and Methods

### 2.1. Sample Collection

Beginning in 2019, an epidemic characterized by paralysis and diarrhea occurred across multiple ostrich farms in the Guangxi Zhuang Autonomous Region, China. According to onsite farm records, the estimated morbidity rate during the epidemic was approximately 80%, and the mortality rate was about 14.79% (Table 1). Tissue samples—including the sciatic nerve, kidney, spleen, liver, heart, brain, and intestine—were collected from 15 affected and deceased ostriches for subsequent viral detection and isolation. Among the 15 samples, all 15 were positive for OsPV, originating from 2 individuals from Guilin, 6 from Xixiangtang (Nanning), 5 from the Economic Development Zone (Nanning), and 2 from Wuzhou farms. A portion of each tissue sample was fixed in formalin (Biosharp, Beijing, China), embedded in paraffin (Biosharp, Beijing, China), sectioned, and stained with hematoxylin and eosin (H&E) (BaSO, Zhuhai, China). All tissue sectioning and hematoxylin and eosin (H&E) staining procedures were commercially outsourced to a professional testing institution (Servicebio, Wuhan, China). The remaining tissue portions were stored at −80 °C for further molecular analyses. Histopathological examination was performed using a Nikon SMZ745 microscope (Nikon Corporation, Tokyo, Japan) (additional details regarding clinical anatomy and histopathology are provided in the Supplementary Materials).

All necropsies were performed by a single investigator (Changting Li), who was blinded to the PCR status of the birds at the time of examination. Samples for PCR were collected during necropsy and analyzed subsequently.

### 2.2. PCR Detection

Tissue samples, including the liver, spleen, and spinal cord, were homogenized in sterile water. Approximately 500 μL of the diluted tissue homogenate was transferred to a 1.5 mL centrifuge tube and centrifuged at 12,000 rpm for 5 min. The supernatant was collected, and DNA extraction was performed using the Bacteria/Virus DNA Extraction Kit (Cwbio, Nanning, China) according to the manufacturer’s instructions. Conventional PCR assays were conducted to screen for nine potential pathogens: duck Tembusu virus (DTMUV), Newcastle disease virus (NDV), *S. Typhimurium*, *Escherichia coli*, duck plague virus (DPV), avian infectious bursal disease virus (IBDV), avian influenza virus (AIV), and ostrich parvovirus (OsPV), goose parvovirus (GPV) using specific primers (Table 2) [24,25]. OsPV-specific primers were designed targeting a conserved region of the Rep gene, based on the publicly available genome sequence of Protoparvovirus sp. isolate HB-2019 (GenBank accession: MT010852.1). 25 μL reaction system: 2 × Rapid Taq Master Mix 12.5 μL, ddH2O 7.5 μL, 2 μL of each primer, and 1 μL of target DNA. PCR reaction procedure: Initial denaturation at 94 °C for 3 min; 30 cycles of denaturation at 94 °C for 30 s, annealing at 52 °C for 30 s, and extension at 72 °C for 45 s; Final extension at 72 °C for 10 min, followed by cooling and maintenance at 4 °C. The amplified PCR products were sequenced to confirm their identity, and the results were analyzed using the NCBI Basic Local Alignment Search Tool (BLAST+ 2.10) to identify sequences with the highest homology.

### 2.3. Whole Genome Amplification of OsPV GX-2020 Strain and Sequencing

To obtain the complete nucleotide sequence of the ostrich parvovirus identified in clinical samples (designated GX-2020), sequence alignments and homology comparisons were performed using reference parvovirus sequences available in GenBank(Release 240). Based on these comparisons, seven pairs of overlapping primers (Table 3) were designed to span the entire viral genome. PCR amplification was conducted using PrimeStar HS DNA polymerase (TaKaRa Biotechnology, Dalian, China). The amplified products were analyzed by 1% agarose gel electrophoresis, gel-purified, and subsequently cloned into the pMD18-T vector (Sangon Biotech, Shanghai, China) for Sanger sequencing. To ensure base-calling accuracy, three independent replicate clones were sequenced for each fragment, and the results were cross-aligned to establish a consensus sequence. Finally, the overlapping sequence fragments were assembled using were commercially synthesized and assembled by Shangong (Shanghai, China) to reconstruct the full-length genome of the OsPV strain.

### 2.4. Phylogenetic and Recombination Analysis

A phylogenetic tree was constructed based on the nucleotide sequences of GPV, MDPV, N-GPV, OsPV, the newly identified GX-2020 strain, and seven additional international waterfowl parvovirus reference strains (Table 4). Multiple sequence alignments and pairwise homology comparisons at both nucleotide and amino acid levels were performed in MEGA 11.0. Phylogenetic reconstruction was conducted using the neighbor-joining method within MEGA 11.0. Node support was determined by bootstrap analysis with 1000 replicates. The resulting phylogenetic tree was visualized and annotated using the Interactive Tree of Life (iTOL V7.6) platform.

**Table 4:** Description of avian parvovirus isolates compared in this study.

GenBank No.	Strains	Collection Year	Country of Origin	Host
OR553572	OsPV GX	2020	China	Ostrich
MT010852	OsPV HB	2019	China	Ostrich
MK281602	OsPV-BJ	2020	China	Ostrich
MK281604	OsPV-YN	2020	China	Ostrich
MK281605	OsPV-YQ	2020	China	Ostrich
MK281603	OsPV-SJZ	2020	China	Ostrich
KM272560	LH	2012	China	Goose
KC996730	YZ99-6	1999	China	Goose
KR091960	YZ	2013	China	Goose
KR091959	WX	2013	China	Goose
MW386079	Yozgat/19	2021	Turkey	Goose
MW386078	Konya/19	2021	Turkey	Goose
MW386077	Corum/19	2021	Turkey	Goose
KR136258	Yan-2	2013	China	Yan goose
EU583391	06-0329	2008	China	Goose
EU583390	82-0321	2008	China	Goose
KC184133	E	2012	China	Goose
KY475562	RC16	2016	China	Goose
MH209633	DY16	2016	China	Goose
MF942876	SQ0412	2017	China	Goose
MH717785	RC70	2017	China	Goose
MH717784	RC45	2017	China	Goose
EU583392	VG32-1	2008	China	Goose
KC996729	SYG61v	2008	China	Goose
HQ891825	GDaGPV	1978	China	Goose
KT598506	GPV-98E	1998	China	Goose
KY511292	FJ	2018	China	Goose
KT598505	GPV-98D15	2012	China	Goose (duck embryo)
KU684472	GPV GER	2015	Poland	ornamental duck
KR029617	G7	2013	China	Muscovy duck
KM093740	MDPV-GX5	2011	China	Muscovy duck
KC171936	SAAS-SHNH	2012	China	Muscovy duck
MH807698	JH10	2010	China	Muscovy duck
MH204100	GDNX	2016	China	Muscovy duck
MH807697	JH06	2006	China	Muscovy duck
KY744743	ZW	2006	China	Muscovy duck
MG932366	YL08	2008	China	Muscovy duck
KX000918	YY	2000	China	Muscovy duck
KC178571	Y	2011	China	Muscovy duck
MF438102	MDE	2015	China	Mule duck
KY069274	LH	2008	China	Mule duck
KU844283	FJ15	2015	China	Mule duck
MH444514	GD	2016	China	Mule duck
KT751090	QH15	2015	China	Peking duck
KT935536	JS1	2015	China	Pekin duck
KT935531	JS1	2015	China	Pekin duck
MF441223	SDHZ1604	2015	China	Cherry Valley ducks
MF441227	AH1605	2016	China	Cherry Valley ducks
MF441222	SDLY1602	2015	China	Cherry Valley ducks
MF441221	SDLY1512	2015	China	Cherry Valley ducks
MF441226	JS1603	2016	China	Cherry Valley ducks
MF441225	AH1606	2016	China	Cherry Valley ducks
KX384726	DPV DS15	2016	China	Cherry Valley ducks
MF441224	SDDY1605	2016	China	Cherry Valley ducks
MH444513	AH	2019	China	Cherry Valley ducks
KY679174	SC16	2015	China	Cherry Valley ducks
KT343253	SDLC01	2015	China	Cherry Valley ducks
MK736656	HuN18	2018	China	Linwu Sheldrake
KU641558	CVSD01	2015	China	Anas platyrhynchos
NC006147	FM	1995	China	Cairina moschata
KY511124	GPV SD	2015	China	Duck
OP265005	VNUA-07	2021	Vietnam	Duck
OP265006	VNUA-26	2021	Vietnam	Duck
OP265007	VNUA-30	2021	Vietnam	Duck
OP265008	VNUA-94	2021	Vietnam	Duck
JF333590	SH	2009	China	Anser
U25749	B	1995	Hungary	Anser
KT232256	FJ01	2012	China	Anser cygnoides(eggs)
KC478066	SHFX1201	2012	China	swan

Potential recombination events were investigated using the Recombination Detection Program (RDP4, version 4.37) with default parameters. Nine detection algorithms—RDP, GENECONV, BootScan, MaxChi, Chimaera, SiScan, Phyl-Pro, LARD, and 3Seq—were utilized to identify recombination events among the waterfowl parvovirus sequences retrieved from GenBank. A recombination event was considered valid and subjected to further validation if it was supported by at least three different detection methods, exhibited a *p*-value < 10^−5^, or had a recombination score exceeding 0.6 [26]. Subsequent validation was performed using SimPlot software 3.5.15 with a window size of 200 base pairs (bp) and a step size of 20 bp.

### 2.5. Statistical Analysis

The statistical and analytical software used in this study included MEGA 11.0, RDP Beta 4.37, iTOL V7.6, and Simplot 3.5.15. All software was operated in accordance with the official user manuals and relevant research guidelines. The criteria for determining the validity of recombination events were based on previous studies to ensure the validity and standardization of the statistical analysis methods [20]. Method for calculating the incidence rate: Morbidity rate = (Number of new cases/Total population during the same period) × 100%. Mortality rate = (Number of deaths/Total population during the same period) × 100% [26].

## 3. Results

### 3.1. Clinical Manifestations and Histopathologic Findings in Ostrich

Ostriches aged approximately 7 to 15 days initially exhibited clinical signs, with disease severity peaking at around 20 days of age. Affected birds gradually developed leg weakness and paralysis, stopped feeding, and became progressively weak and emaciated. Prolonged immobility appeared to impair intestinal motility and was associated with intestinal obstruction and death in severe cases.

To characterize pathological changes in the affected ostriches, gross anatomical observations were performed. The intestinal mucosa was markedly congested and edematous, with prominent congestion of the mesenteric vessels (Figure 1A). The intestinal wall was thin and translucent, and the intestinal contents were pasty and mixed with bubbles and blood streaks (Figure 1D). The liver was markedly enlarged and dark red to reddish-brown, with a tense liver capsule and yellow thoracic fluid (Figure 1B). Focal hemorrhagic and necrotic lesions were visible in the leg muscles, with local deep brown to black discoloration of the muscle surface, consistent with severe muscle injury (Figure 1C). The brain showed congestion and edema, suggesting possible nervous system involvement, although direct viral invasion was not demonstrated (Figure 1E). The heart exhibited extensive hemorrhagic lesions, with multiple dark red to purple areas and abnormal tissue texture, suggesting myocardial hemorrhage and possible necrosis (Figure 1F). Collectively, these gross findings indicate a systemic disease process involving the digestive, hepatic, muscular, nervous, and cardiovascular systems. However, because healthy control ostriches were unavailable and no quantitative lesion scoring was performed, these findings should be interpreted as descriptive pathological observations rather than definitive evidence of OsPV-induced lesions.

Histopathological examination of liver (Figure 2A,D), spleen (Figure 2B,E), and duodenum (Figure 2C,F) sections from deceased ostriches showed disruption of normal tissue architecture, indistinct structural boundaries, disorganized cellular arrangement, red blood cell infiltration, compromised cell membranes, pyknotic nuclei, and inflammatory cell infiltration. Because only hematoxylin and eosin-stained sections were available, inflammatory cell subtypes could not be reliably distinguished without immunohistochemistry.

### 3.2. Detection of the OsPV and Complete Genome Sequencing

To identify the pathogen responsible for the epidemic on the studied ostrich farm in the Guangxi Zhuang Autonomous Region, PCR was conducted to screen for pathogens potentially causing paralysis and diarrhea. The results indicated that the samples were positive for ostrich parvovirus (OsPV) but not detected for GPV, DTMUV, NDV, *S. Typhimurium*, *E. coli*, DPV, IBDV, and AIV. The tissue distribution of positivity was as follows: sciatic nerve (15/15 positive), kidney (15/15), spleen (15/15), liver (15/15), heart (15/15), brain (15/15), and intestine (15/15). The newly identified virus was designated OsPV GX-2020 (Figure 3). Seven overlapping PCR fragments covering the full length of the viral genome were successfully obtained. The complete nucleotide sequence of the GX-2020 genome has been deposited in GenBank (accession number: OR553572). Genetic analysis demonstrated that the ostrich parvovirus GX-2020 strain shared the highest sequence homology with the ostrich parvovirus HB-2019 isolate, followed by goose parvovirus (GPV).

### 3.3. Genomic Characterization and Homology Analysis

The complete genomic sequence of the OsPV (GX-2020) was determined and deposited in the GenBank database (accession number: OR553572). The genome is 5046 nucleotides (nt) in length and is flanked by two inverted terminal repeats (ITRs) at the 3′ and 5′ ends. The genome contains two major open reading frames (ORFs): the left ORF encodes two non-structural replication (Rep) proteins, while the right ORF encodes three structural capsid (Cap/VP) proteins, exhibiting a genomic organization characteristic of classical goose and Muscovy duck parvoviruses.

Sequence homology comparisons revealed that the full-length GX-2020 genome shares 99.90% nucleotide identity with the GPV strain DY16, but exhibits lower similarity to the NGPV strain HuN18 (96.90%), the Vietnamese GPV strain VNUA (95.41%), and the MDPV strain FJ15 (86.40%) (Table 5). At the genetic level, the Rep gene of GX-2020 displayed high nucleotide sequence identity (96.55–99.94%) with classical GPV strains, but lower similarity (83.30–83.70%) to classical MDPV strains (Table 5). Similarly, nucleotide sequence comparisons of the VP1 gene showed that GX-2020 shared the highest percentage of identity with classical GPV strains (95.42–99.86%), whereas its identity with classical MDPV strains was significantly lower (87.4–92.6%) (Table 5).

**Table 5:** Comparison of nucleotide of whole genome, NS sequence, and VP sequence between OsPV GX-2020 and other waterfowl parvoviruses.

Sequence Name	Classic GPV	N-GPV	MDPV	Vietnamese GPV
Whole genome	93.99–99.90%	93.70–96.90%	83.30–86.40%	95.30–95.41%
VP nucleotide sequences	95.42–99.86%	95.51–96.43%	87.4–92.6%	95.60–96.06%
NS nucleotide sequences	96.55–99.94%	96.70–99.71%	83.30–83.70%	96.99–97.17%

Note: All homology values in Table 5 represent fixed pairwise nucleotide identities between individual viral strains. No biological replicates or parallel experimental groups were involved in sequence alignment. Only the identity range and extreme values are presented herein.

### 3.4. Phylogenetic Analysis

A phylogenetic analysis was conducted using the complete genome sequences of the novel OsPV and reference parvoviruses available in GenBank, including classical GPV, MDPV, MDGPV, and N-GPV. As illustrated in Figure 4, whole-genome phylogenetic analysis classified the 75 analyzed parvovirus strains into four main clusters, with the newly identified OsPV and the HB-2019 strain (an ostrich parvovirus isolated in Hebei in 2019) forming a distinct branch. Further phylogenetic and comparative analyses confirmed that OsPV represents a unique sub-lineage within the GPV group (Figure 4). Notably, there was a significantly greater genetic distance between OsPV and MDGPVs, as well as between OsPV and N-GPVs, compared to its distance to classical GPVs.

Multiple sequence alignment revealed that, compared to the virulent GPV strain B and the vaccine strain SYG61v, OsPV shares the same deletions in the 3′-ITR and 5′-ITR regions as those observed in the GPV strain DY16 and the HB-2019 isolate (Figure 5A). Furthermore, comparative analyses among OsPV GX-2020, HB-2019, virulent GPV strain B, and the vaccine strain SYG61v identified amino acid mutations in the capsid protein (Figure 5B).

### 3.5. Recombination Analysis

In the GX-2020 strain, Simplot software was used to analyze the recombination; two potential recombination events were detected. RDP4 was used to further analyze, which revealed breakpoints located in two areas of the VP1 region ((nucleotide positions 2475 and 2726, 3805 and 4318; Figure 6). The recombination analysis results show that GX-2020 is the product of recombination between the virulent B strain and the vaccine strain SYG61v of GPV.

## 4. Discussion

Ostrich farming represents a unique and economically significant sector of animal husbandry, playing a vital role in the agricultural development of South China. However, since 2020, farms in the Guangxi region have experienced recurrent outbreaks of a disease characterized by paralysis and diarrhea in ostriches. Empirical treatments, including antibiotics, antiviral therapeutics, and antibody administration, proved ineffective. In this study, we investigated the potential etiological agents responsible for these outbreaks. We consistently detected ostrich parvovirus (OsPV) in clinical samples collected across multiple farms, indicating the widespread prevalence of this virus in regional ostrich breeding facilities. Screening for other common viral and bacterial pathogens yielded negative results, which is consistent with previous reports [15]. Based on our recommendations, farmers in Hengzhou City (Guangxi Zhuang Autonomous Region) and surrounding areas administered a commercial goose parvovirus (GPV) vaccine (strain SYG61v) to healthy ostriches. Following vaccination, the incidence of characteristic clinical signs, such as paralysis and recumbency, decreased significantly, with the rate of leg paralysis dropping from 40% to 0%. Although definitive causation cannot be established due to the lack of an experimental animal model to fulfill Koch’s postulates, the clinical efficacy of GPV vaccines in preventing the disease, combined with consistent clinical presentations, may suggest an association between OsPV infection and the observed paralysis. We emphasize that this observation was not obtained from a controlled study; information regarding flock size, standardized case definition, observation duration, and parallel control groups was not systematically recorded. Consequently, confounding factors—including concurrent improvements in farm management and potential shifts in the prevalence of other pathogens—cannot be excluded. Whether the reduction in clinical signs was attributable to vaccination or to these co-occurring changes remains undetermined. Controlled challenge and vaccination experiments are required to validate this preliminary field observation and to evaluate whether OsPV plays a causal role in the observed paralysis.

Post-mortem examinations of mortalities associated with this outbreak revealed distinct pathological changes in the liver, intestines, and brain. This presentation contrasts with a 2020 study, which reported an absence of gross lesions in the majority of affected ostriches. Conversely, a 2022 study documented hemorrhaging in the leg muscles, thymus, and bursa of Fabricius, alongside renal tubular degeneration associated with interstitial nephritis and severe hepatitis. That study also noted edema and swelling in the knee joints [23]. Although previous studies have documented renal tissue involvement, notable discrepancies exist between their pathological findings and those observed in our study. These variations may be attributed to differences in viral strains, geographical environments, and flock management practices. Furthermore, we did not detect secondary pathogenic bacteria in our clinical samples. This absence may be due to the extensive administration of antibiotics by farmers prior to necropsy, which likely suppressed bacterial loads and impeded our ability to isolate concurrent bacterial pathogens. Nevertheless, the characteristic paralysis observed in the affected ostriches in our study remains highly consistent with the clinical presentations reported during the 2020 and 2022 outbreaks [23,27].

To our knowledge, this is the first study to report the detection of OsPV DNA sequences in commercial ostrich farms in South China. Phylogenetic analysis demonstrated that this identified virus is closely related to classical goose parvovirus (GPV). The full-length genome of the OsPV strain GX-2020 is 5046 nucleotides in length and exhibits the highest nucleotide sequence identity with the classical virulent GPV strain DY16, followed by the ostrich parvovirus isolate HB-2019. Given the geographic proximity of the Guangxi Zhuang Autonomous Region to Vietnam, we conducted an evolutionary analysis comparing GX-2020 with Vietnamese waterfowl parvovirus strains to assess the potential for cross-border transmission. However, this analysis revealed a distant genetic relationship, suggesting that GX-2020 is most likely an endemic strain rather than a recently imported variant [15]. Phylogenetic analysis also revealed that the newly identified virus GX-2020 strain clusters with ancestral strains. Similar to isolates from other geographical regions, strains originating from the same locale cluster into distinct subclades. This pattern suggests a common evolutionary origin, followed by regional divergence after the virus was introduced to specific areas. This dissemination may have occurred via the transport of breeding birds from Xinjiang to South China—a prominent waterfowl farming region—ultimately resulting in cross-species transmission. Although the precise factors driving this cross-species spillover remain unclear, subsequent domestic ostrich trade likely facilitated the spread of GPV to northern China.

Recombination represents a critical mechanism of viral evolution. Novel variant strains emerging from recombination events often exhibit expanded host tropism and an enhanced capacity for immune evasion, which can precipitate disease outbreaks even in vaccinated populations. Previous studies have demonstrated that recombination plays a pivotal role in parvovirus evolution, with substantial evidence documenting such events among waterfowl parvoviruses. The GPV genome comprises two open reading frames (ORFs) flanked by inverted terminal repeat (ITR) structures. The ORFs encode non-structural proteins (NS1 and NS2) and structural proteins (VP1, VP2, and VP3) [28]. VP2 and VP3 are translated internally from VP1, which means that VP1 contains all the amino acid sequences for VP2 and VP3 [29]. VP3 is the main structural protein of waterfowl parvoviruses, and it is crucial for determining which hosts the virus can infect and how harmful it is [30]. The Rep protein has multiple functions: it helps replicate the virus genome and package virus particles by interacting with ITRs [31]. Compared to the GPV vaccine strain SYG61v and the virulent strain B, the OsPV genome possesses two deletions within its inverted terminal repeat (ITR) regions. Although these deleted nucleotides were located at base-pairing positions and did not disrupt the hairpin structure formation of the remaining sequence, the deletions shortened the spatial distance between adjacent transcription factor binding sites. Whether these ITR nucleotide deletions influence transcriptional efficiency and contribute to the virulence of the GX-2020 remains to be further investigated.

In this study, recombination analysis using RDP4 and SimPlot software identified two distinct recombination events in the OsPV GX-2020 genome. Specifically, segments of the VP gene (nucleotide positions 2475–2726 and 3805–4318) exhibit the highest sequence identity with the vaccine strain SYG61v, whereas the remainder of the genome shares high similarity with the virulent GPV strain B. SYG61v is an attenuated GPV vaccine strain conventionally used to protect young waterfowl from GPV infection. In contrast, strain B is one of the earliest European GPV isolates, initially identified in Hungary in 1967 [32]. Our analysis confirmed that SYG61v and strain B both participated in the recombination occurring within the VP3 region of GX-2020. These results strongly suggest that OsPV GX-2020 is most likely a recombinant GPV strain, closely resembling other ostrich parvoviruses reported across China [27]. These findings indicate that GPV strains of distinct geographical origins jointly contributed to the emergence of the recombinant GX-2020. Viral recombination likely acts as a key driver for such cross-species transmission events; however, further research is urgently needed to fully elucidate the underlying mechanisms. Notably, the recombinant fragments are located within the VP3 capsid coding region (which harbors major conformational epitopes and surface-exposed variable loops), and the resulting chimeric VP3 may affect immune evasion and cross-species transmission by altering epitope recognition and receptor binding; however, this functional hypothesis requires experimental validation [33].

Several limitations to this study should be acknowledged. First, due to logistical constraints during on-site sampling, we were unable to collect samples from healthy ostriches on the affected farms to serve as negative controls. Second, the absence of a systematic quantitative histopathological analysis may confound the differentiation between virus-induced lesions and post-mortem autolysis. Third, during PCR screening, standard reference strains for all targeted pathogens were unavailable at the time, precluding the use of routine positive controls and potentially limiting the definitive interpretation of negative diagnostic results. Our pathogen screening panel mainly targeted common waterfowl parvoviruses and bacterial pathogens. Several potential viral pathogens associated with ostrich paralysis and diarrhea, including avian reovirus, astrovirus, adenovirus, circovirus, and bornavirus, were not included, leading to incomplete pathogen coverage. Fourth, although all 15 deceased birds were PCR-positive for OsPV, only one sample underwent whole-genome sequencing, whereas the remaining positive samples underwent only routine sequencing and BLAST analysis, not whole-genome sequencing. Therefore, this single complete genome should be regarded only as a representative sequence; it cannot represent all 15 PCR-positive birds from multiple farms, nor can it reflect viral genetic diversity across farms or cases. Fifth, the lack of animal challenge experiments prevents definitive confirmation of the causal relationship between OsPV and the paralytic disease hypothesis. Furthermore, viral genome sequences were obtained via PCR-based genome walking using primers designed from published reference sequences. While standard practice, this method inherently relies on existing genetic data and may fail to amplify highly divergent or novel genomic regions.

Attempts to isolate the novel virus using various embryonic systems—including chicken, duck, goose, and ostrich embryos—were uniformly unsuccessful (Supplementary Materials). This failure is likely attributable to low viral loads in the initial clinical samples and underscores the broader technical challenges associated with isolating novel parvoviruses from atypical hosts. Consequently, there is an urgent need to optimize isolation protocols and develop alternative in vitro cultivation methods. In this regard, goose embryo fibroblasts (GEF) have been successfully used for the isolation and propagation of GPV, with reports demonstrating that GPV can be adapted to GEF through serial passaging, accompanied by increased viral titers and evident cytopathic effects. Additionally, Muscovy duck embryo fibroblasts (MDEF) have also been employed for GPV adaptation. However, the applicability of these cell systems to OsPV remains unknown, and their potential for OsPV isolation warrants further investigation [34,35,36].

Interestingly, while the commercial GPV vaccine demonstrated clinical efficacy, the long-term management of this disease will require the development of customized vaccines tailored to the specific antigenic profiles of ostrich-origin isolates. The observed cross-protection suggests that OsPV and GPV share conserved antigenic epitopes, further supporting the classification of OsPV within the GPV lineage. However, definitive causation between OsPV and the observed disease cannot be established from our observational data alone, as confounding variables—such as concurrent improvements in flock management or fluctuations in the prevalence of other pathogens—cannot be entirely excluded. Rigorous experimental challenge studies are required to validate this hypothesis.

Future studies should employ reverse genetics to construct full-length infectious clones of ostrich-origin GPV, enabling the identification of specific genetic determinants of virulence through targeted mutagenesis. It is equally critical to establish an in vivo animal model (e.g., inoculation trials in goslings or SPF ostrich chicks, along with in vitro CPE assays on primary ostrich embryonic fibroblasts or other permissive cell lines to visualize virus-induced changes, purify the virus, and determine titers) to definitively confirm OsPV-induced paralysis and to elucidate the underlying pathogenesis—specifically, to determine whether the virus exhibits direct neurotropism or induces tissue damage via immune-mediated mechanisms. Furthermore, the rapid dissemination of novel antigenic variants, driven by ongoing mutation and recombination, underscores the necessity for continuous molecular epidemiological surveillance and the regular updating of disease control strategies.

## 5. Conclusions

In summary, this study highlights the significant threat that ostrich parvovirus (OsPV) poses to the ostrich farming industry in southern China. Our findings indicate that this newly identified virus emerged via a recombination event involving classical GPV strains, facilitating cross-species transmission, and is strongly associated with the observed paralytic disease. Although we cannot yet definitively confirm that OsPV is the sole etiological agent, the available clinical and molecular evidence supports an association between OsPV and ostrich morbidity. This study emphasizes the necessity for continued research to elucidate the precise pathogenic mechanisms underlying these paralytic symptoms and to develop targeted preventive measures. Furthermore, these findings call for heightened awareness among agricultural stakeholders regarding the potential impact of OsPV. Implementing stringent biosecurity measures and continuous surveillance will be essential to control viral transmission, safeguard ostrich populations, and protect the economic viability of the regional agricultural industry.

## Figures and Tables

**Figure 1:**
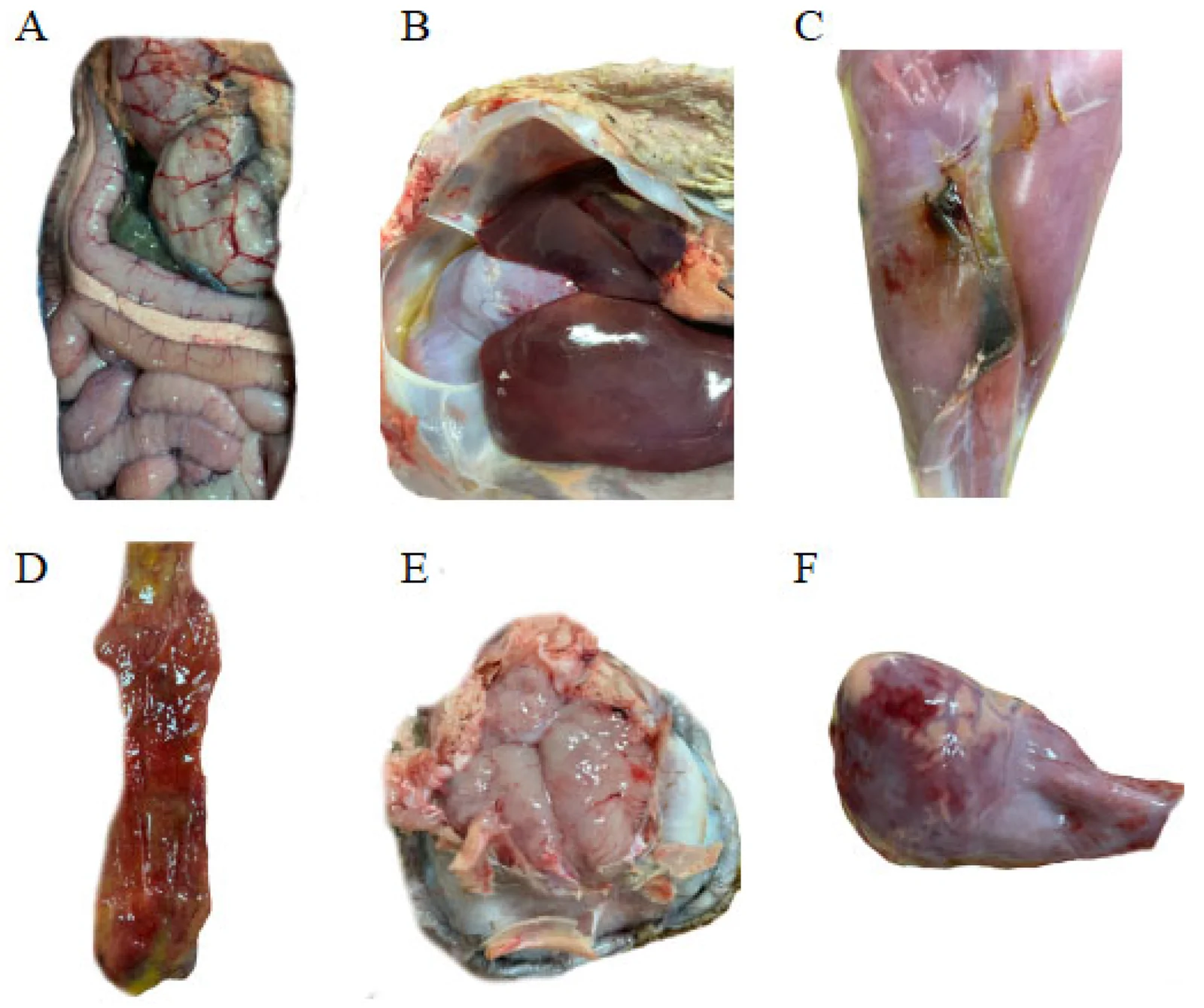
Gross anatomical findings in a deceased ostrich. (**A**) Overall abdominal intestinal tract; (**B**) liver; (**C**) leg muscles; (**D**) intestine; (**E**) brain; (**F**) heart.

**Figure 2:**
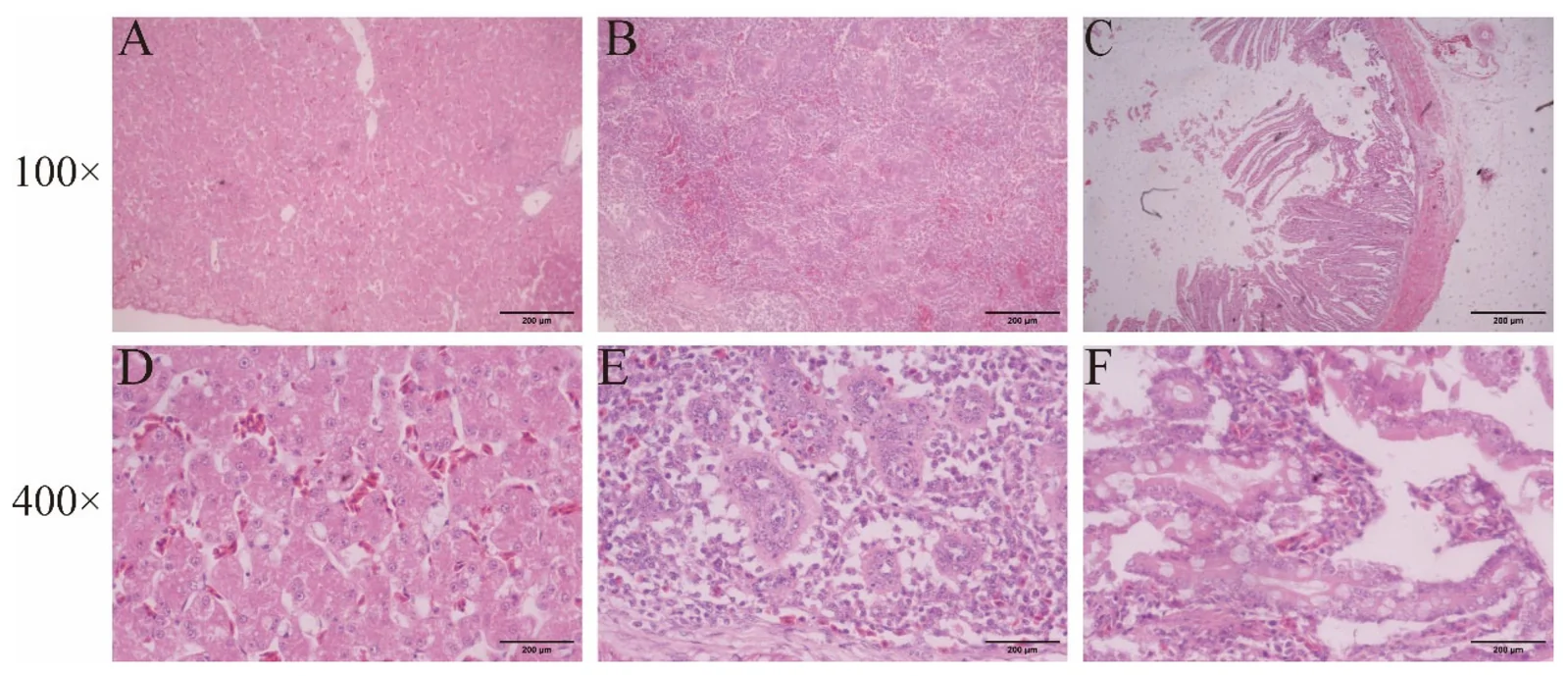
Histopathological sections of tissues from deceased ostriches. (**A**,**D**) Liver; (**B**,**E**) spleen; (**C**,**F**) duodenum. Original magnification: 100× (**A**–**C**) and 400× (**D**–**F**).

**Figure 3:**
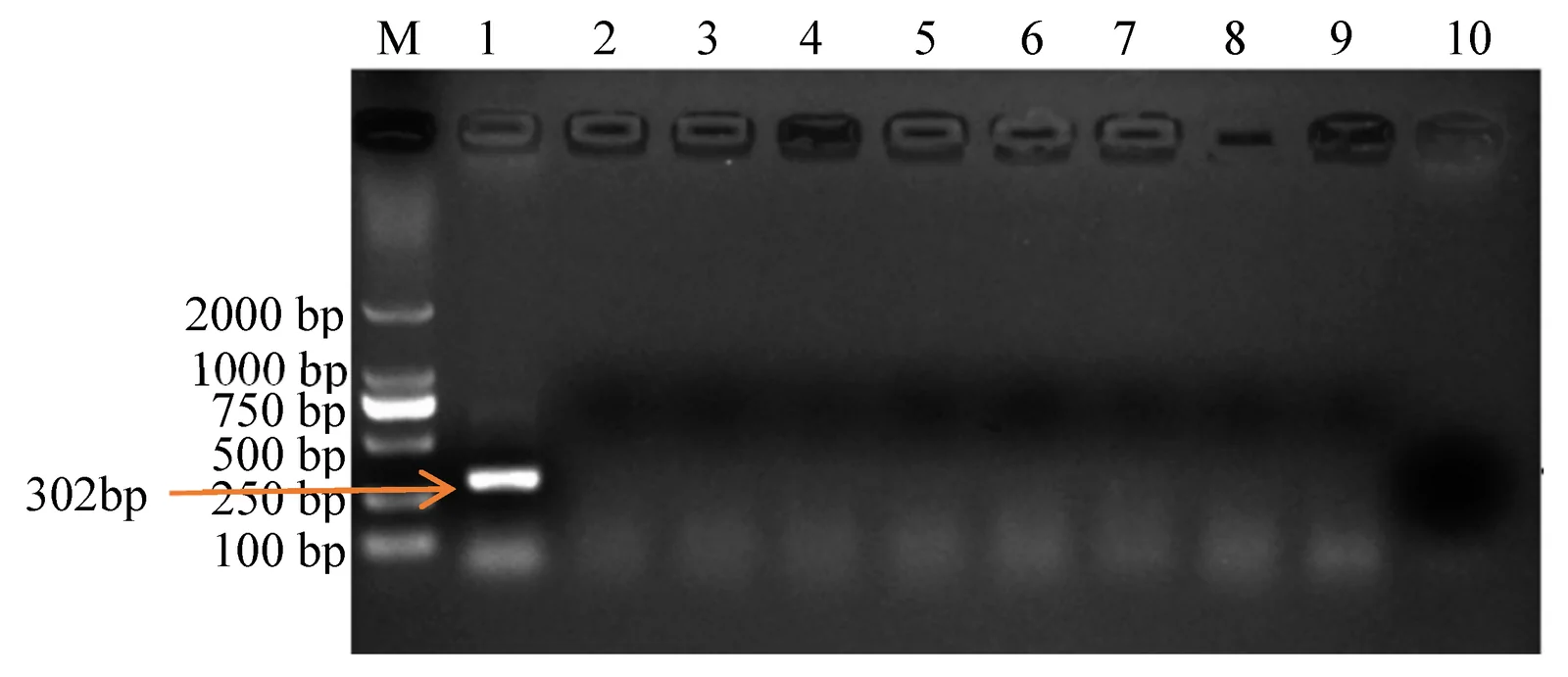
PCR amplification of different agents and complete genome of ostrich parvovirus. Nine potential pathogens were detected with corresponding primers, including Lane 1: OsPV; Lane 2: GPV; Lane 3: DTMUV; Lane 4: NDV; Lane 5: *S. Typhimurium*; Lane 6: *E. coli*; Lane 7: DPV; Lane 8: IBDV; Lane 9: AIV; Lane 10: blank control.

**Figure 4:**
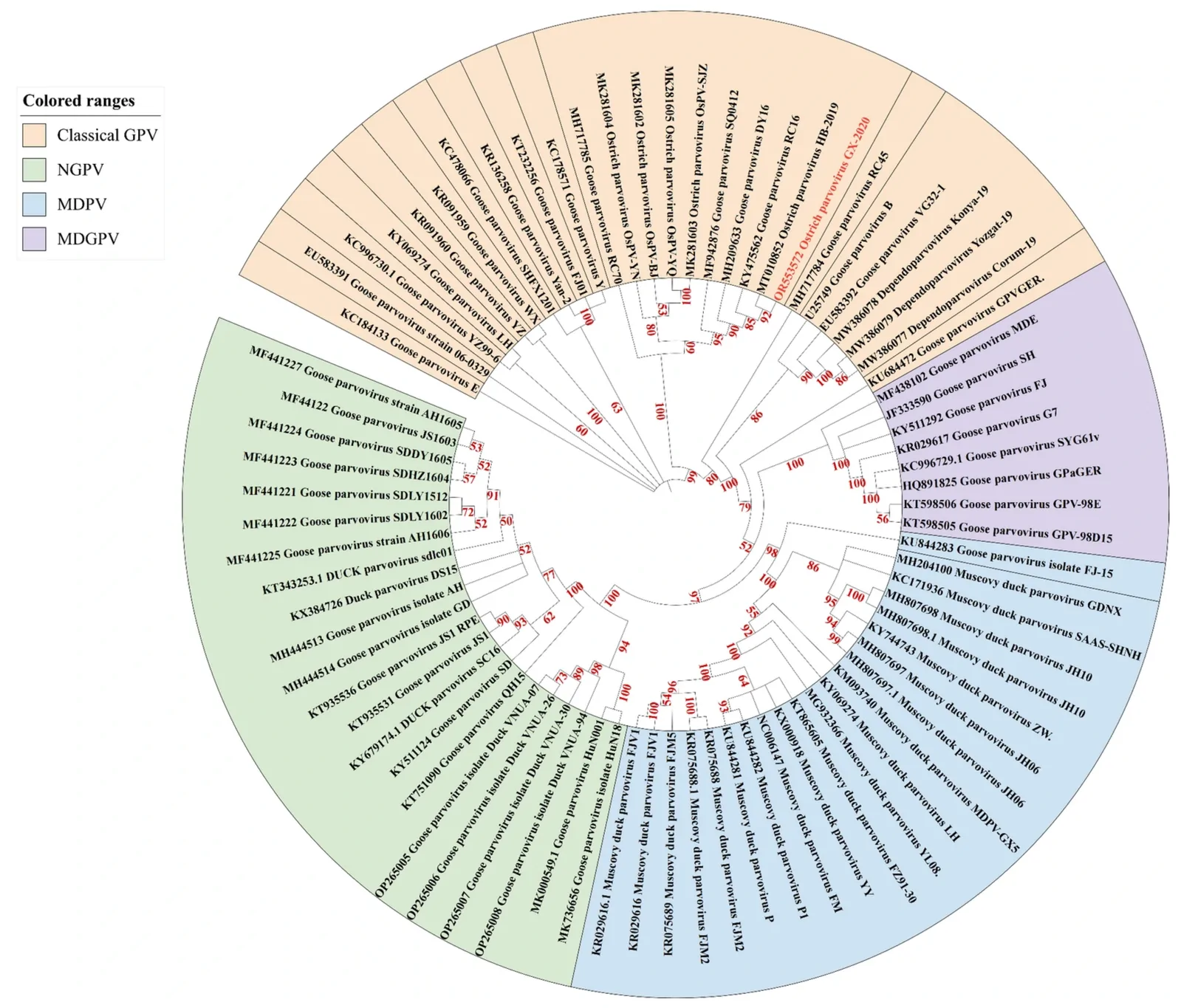
Phylogenetic analysis of OsPV GX-2020 (red font) and other waterfowl parvovirus isolates available in the GenBank database based on the complete genomic sequences. MDPV in this study is indicated with a blue background, classic GPV is indicated with a orange background, N-GPV is indicated with a green background, and MDGPV is indicated with a purple background. Reference sequences obtained from GenBank are indicated by their accession numbers and strain names. The neighbor-joining method in MEGA 11.0 was used for the construction of the phylogenetic tree.

**Figure 5:**
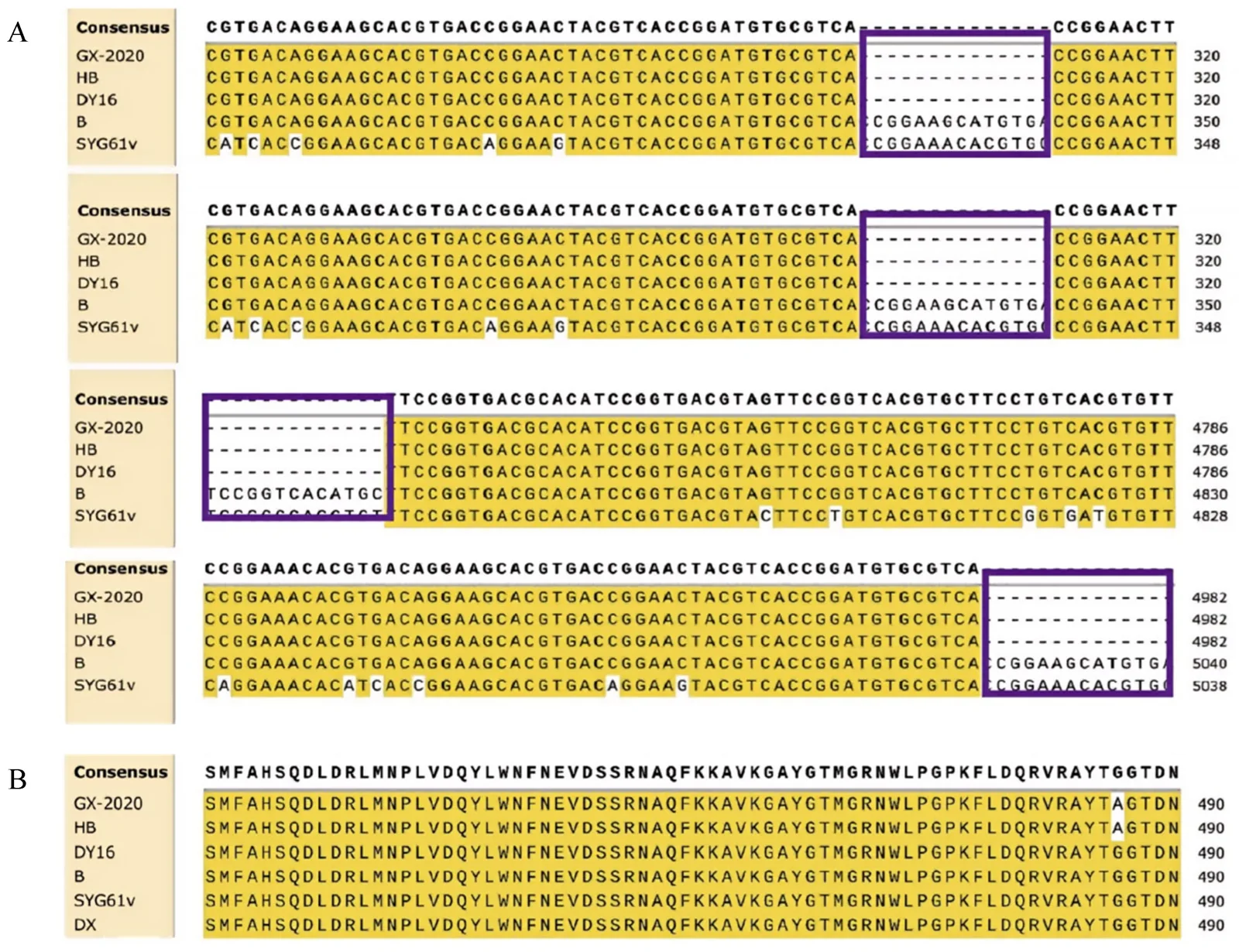
Sequence alignments of the inverted terminal repeats (ITRs) of the OsPV GX-2020. (**A**): Sequence alignments of the ITRs of the OsPV GX-2020 and GPVs (SYG61v, DY16, and B strain). (**B**): Sequence alignments of the amino acid of the OsPV GX-2020 and GPVs (SYG61v, DY16, and B strain).

**Figure 6:**
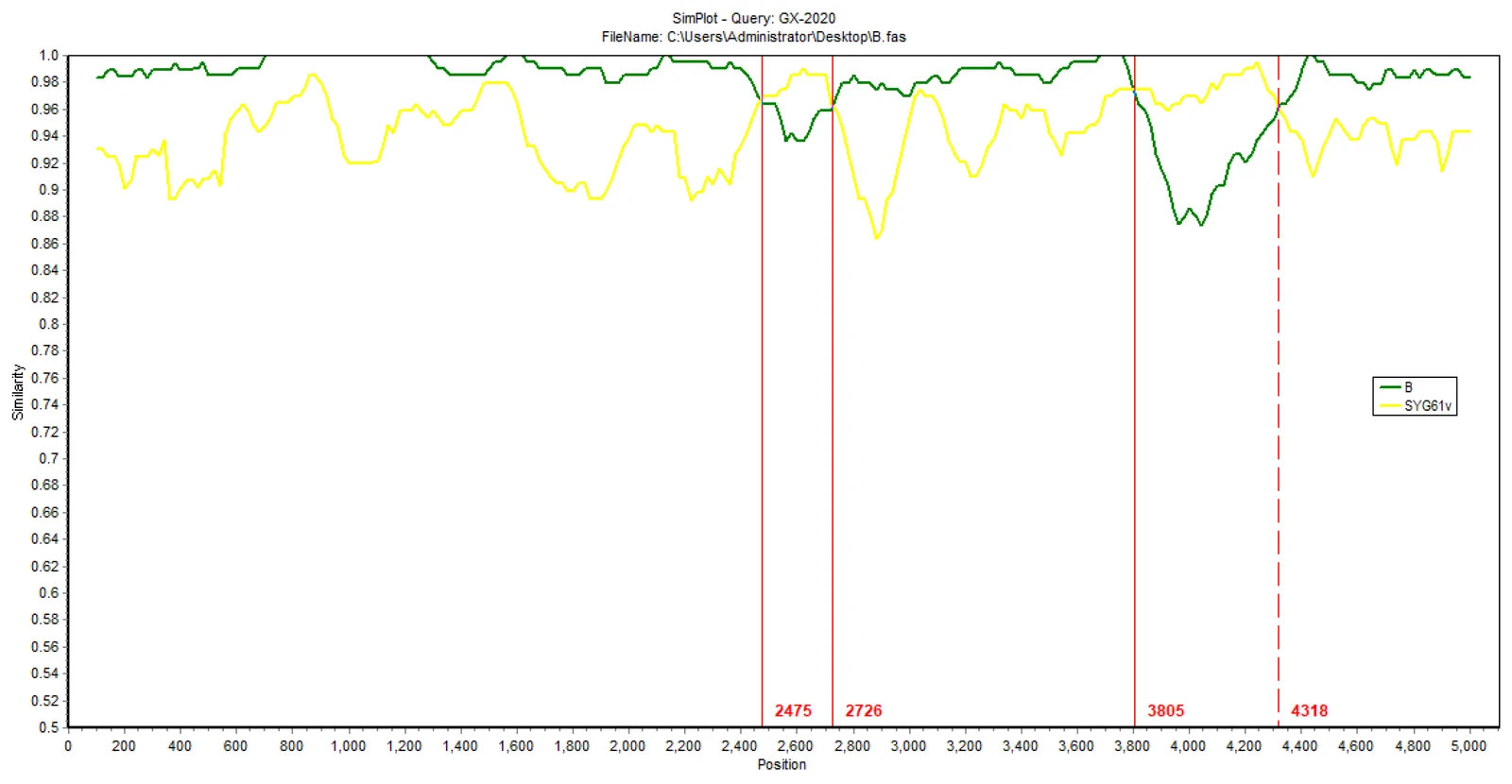
Genome recombination analysis of the OsPV GX-2020 strain. Recombination analysis was calculated using the Simplot 3.5.1 software. This image was automatically generated by the software. The complete genome of OsPV GX-2020 was chosen as the query sequence. Yellow line: SYG61v, green line: B strain. Recombination breakpoints are shown with red lines, and the locations are shown at the bottom. The background color of the major parental regions is white. Y-axis: Similarity score, X-axis: nucleotide position.

**Table 1:** Morbidity and mortality of ostriches in different farms.

Date	Location	Flock Size	Age at Onset	Cases	Deaths
November 2019	Lingchuan County, Guilin	100	30 days	76	10
November 2019	Xixiangtang District, Nanning	200	20 days old	165	36
December 2019	Teng County, Wuzhou	80	30–60 days	61	10
August 2020	Nanning ETDZ	100	30 days	79	15

Note: These values are field estimates provided by farm managers rather than values calculated from samples collected in the present study. Due to sampling constraints, only dead ostriches were available for our investigation; therefore, we are unable to calculate true morbidity from our sample set.

**Table 2:** Primers for pathogen PCR detection.

Primers	Nucleotide Sequence (5′ → 3′)	Length of Products (bp)
GPV-Forward	AAACTTACTGAGCCCGTTCCTG	659
GPV-Reverse	GCGACGCTGTCTGCTTTATTGA	
AIV-Forward	CGTAGACGCTTTGTCCAGAATGC	428
AIV-Reverse	GTCCTCATTGCCTGCACCATC	
DPV-Forward	TATAGCACAACAACCTCAG	491
DPV-Reverse	GCCCCTTTCGTACTGTGAG	
DTMUV-Forward	GGCTTGTTTGGAAAAGG	514
DTMUV-Reverse	TTCCAGAGTACTTCACTG	
NDV-Forward	ATGGGCYCCAGAYCTTCTAC	535
NDV-Reverse	CTGCCACTGCTAGTTGTGATAATCC	
IBDV-Forward	ATGGCAAGACTGTAACGAATG	731
IBDV-Reverse	GCGGTCAGCCCAATAGTT	
*E. coli*-Forward	GACAAAGCCATTGAATTGTTGA	414
*E. coli*-Reverse	GGTGTAGAAGAGATCGGTCTGG	
*S. Typhimurium*-Forward	GCATTATCGATCAGTACCAGCCGTCT	331
*S. Typhimurium*-Reverse	TCGCACCGTCAAAGGAACCGTAAAGC	
OsPV-Forward	AGAAGCAGGAACAATTACCAG	302
OsPV-Reverse	CTTTCAGATTCCGCCACGAG	

**Table 3:** Primers for amplification of Ostrich Parvovirus sequences.

Primers	Nucleotide Sequence (5′ → 3′)	Length of Products (bp)
OsPV-1-F	ATCTACCGATGGTGACCTTG	713
OsPV-1-R	GTCCAGGACAGGGTCTTTTC	
OsPV-2-F	CTGAGAACCGAGAGAGCTACAG	741
OsPV-2-R	GTTCATTCGTTCGGACTTTG	
OsPV-3-F	AAATTGGAGCCTTCTTTTGG	712
OsPV-3-R	AGCCAGGAAGTACAAAGCCC	
OsPV-4-F	ATGGGTCGTATGATTGTGC	1082
OsPV-4-R	CTGCGTTGTGACTTCTTTGA	
OsPV-5-F	GTAATGCCTCGGGAAATTGG	711
OsPV-5-R	ATTGAGCATTTCTGCTGCTG	
OsPV-6-F	TTTCCATAGCATGTTCGCTC	704
OsPV-6-R	CTGCAGGCACTGGTGTATTC	
OsPV-7-F	ATGGCAAGACTGTAACGAATG	731
OsPV-7-R	GCGGTCAGCCCAATAGTT	

## Data Availability

The original contributions presented in this study are included in the article/Supplementary Material. Further inquiries can be directed to the corresponding authors.

## Supplementary Materials

The following supporting information can be downloaded at: https://www.mdpi.com/article/10.3390/microorganisms14102168/s1, Figure S1. Representative embryo lesions after inoculation. (A) Chicken embryo; (B–C) duck embryos; Figure S2. Transmission electron micrograph of the virus.
