# Supplementary material for: Genomic Characterization and Putative Recombination Signals of Ostrich-Associated Parvovirus in South China
Source: Microorganisms. 2026 Sep 25;14(10):2168. doi: 10.3390/microorganisms14102168 (PMC13707664; doi:10.3390/microorganisms14102168)
Supplement: Supplementary file 1 [file microorganisms-14-02168-s001.zip › microorganisms-4550755-supplementary.pdf]

Supplementary

1.Embryo inoculation assay

Tissue samples, including liver, spleen, pancreas, brain, and spinal cord, were collected from deceased ostriches. Samples were homogenized with three volumes of PBS and centrifuged at 12,000 rpm for 5 min. The supernatant was filtered through a 0.22-μm filter, and 0.5 mL of filtrate was inoculated into the allantoic cavity of 8-day-old SPF duck embryos. After inoculation, embryos were incubated at 37 °C and candled twice daily. Embryos that died within 24 h post-inoculation were discarded. The remaining embryos were observed for 7 consecutive days, and blind passages were performed three times. Allantoic fluid was collected aseptically, and gross pathological changes in the embryos were examined.

Following allantoic cavity inoculation with tissue filtrate, embryo death began on day 4 post-inoculation. By days 6-7, the embryo mortality rate reached approximately 70%. Dead embryos showed thickened allantoic membranes and severe embryonic edema (Figure S1). These observations suggest that the inoculated filtrate induced embryo lesions; however, they do not by themselves prove productive OsPV replication or establish a purified viral isolate.

A B C

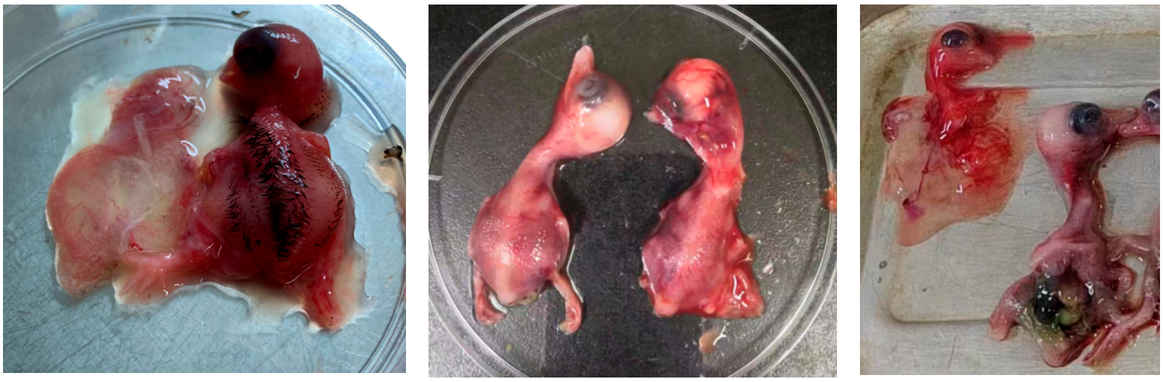

Figure S1. Representative embryo lesions after inoculation. (A) Chicken embryo; (B-C) duck embryos

2. Electron Microscopy Observation

Duck embryo allantoic fluid was collected, inactivated with 0.4% formaldehyde, and submitted to Beijing Lianjing Kechuang Technology Co., Ltd. for transmission electron microscopy. After negative staining, non-enveloped virus-like particles approximately 20-25 nm in diameter were observed in the allantoic fluid samples (Figure S2). Because these observations were not combined with immunogold labeling or additional replication assays, the particles should be interpreted as virus-like particles compatible with parvovirus morphology rather than definitive proof of purified OsPV isolation.

15000 ×

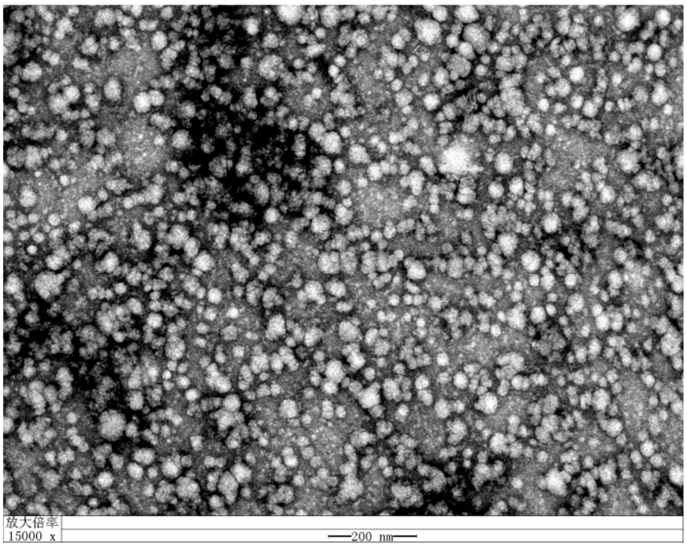

Figure S2 Transmission electron micrograph of the virus
